# Nanocellulose: a bioadsorbent for chemical contaminant remediation

**DOI:** 10.1039/d0ra08005e

**Published:** 2021-02-12

**Authors:** Mohd Nor Faiz Norrrahim, Noor Azilah Mohd Kasim, Victor Feizal Knight, Muhammad Syukri Mohamad Misenan, Nurjahirah Janudin, Noor Aisyah Ahmad Shah, Norherdawati Kasim, Wan Yusmawati Wan Yusoff, Siti Aminah Mohd Noor, Siti Hasnawati Jamal, Keat Khim Ong, Wan Md Zin Wan Yunus

**Affiliations:** Research Centre for Chemical Defence, Universiti Pertahanan Nasional Malaysia Kem Perdana Sungai Besi 57000 Kuala Lumpur Malaysia; Department of Chemistry and Biology, Centre for Defence Foundation Studies, Universiti Pertahanan Nasional Malaysia Kem Perdana Sungai Besi 57000 Kuala Lumpur Malaysia azilah@upnm.edu.my; Department of Chemistry, College of Arts and Science, Yildiz Technical University, Davutpasa Campus 34220 Esenler Istanbul Turkey; Department of Physics, Centre for Defence Foundation Studies, Universiti Pertahanan Nasional Malaysia Kem Perdana Sungai Besi 57000 Kuala Lumpur Malaysia; Research Centre for Tropicalisation, Universiti Pertahanan Nasional Malaysia Kem Perdana Sungai Besi 57000 Kuala Lumpur Malaysia

## Abstract

Chemical contaminants such as heavy metals, dyes, and organic oils seriously affect the environment and threaten human health. About 2 million tons of waste is released every day into the water system. Heavy metals are the largest contributor which cover about 31% of the total composition of water contaminants. Every day, approximately 14 000 people die due to environmental exposure to selected chemicals. Removal of these contaminants down to safe levels is expensive, high energy and unsustainable by current approaches such as oxidation, biodegradation, photocatalysis, precipitation, reverse osmosis and adsorption. A combination of biosorption and nanotechnology offers a new way to remediate these chemical contaminants. Nanostructured materials are proven to have higher adsorption capacities than the same material in its larger-scale form. Nanocellulose is very promising as a high-performance bioadsorbent due to its interesting characteristics of high adsorption capacity, high mechanical strength, hydrophilic surface chemistry, renewability and biodegradability. It has been proven to have higher adsorption capacity and better binding affinity than other similar materials at the macroscale. The high specific surface area and abundance of hydroxyl groups within lead to the possible functionalization of this material for decontamination purposes. Several research papers have shown the effectiveness of nanocellulose in the remediation of chemical contaminants. This review aims to provide an overview of the most recent developments regarding nanocellulose as an adsorbent for chemical contamination remediation. Recent advancements regarding the modification of nanocellulose to enhance its adsorption efficiency towards heavy metals, dyes and organic oils were highlighted. Moreover, the desorption capability and environmental issue related to every developed nanocellulose-based adsorbent were also tackled.

## Abstract 2

The adsorption and desorption of contaminants by nanocellulose.

## Graphical abstract

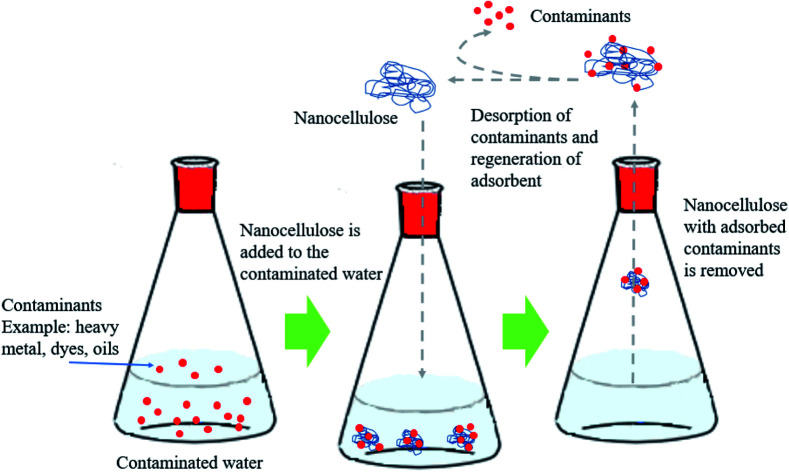

## 1. Introduction

The industrial revolution has positively contributed to the growth of the world's economy and towards sociological advancement. Ever increasing human activities in concert with the industrial revolution and its consequences have resulted in the occurrence of chemical contamination of the environment in many parts of the world. Thus in order to reduce the magnitude of the effects from chemical contamination, the management of contaminated sites represents an important, urgent and ever increasing need.^1^ Heavy metals, dyes, dissolved organic solvents, pesticides and herbicides are among the common chemical contaminants that usually are found in the environment especially in both industrial wastewater and groundwater.^2^ The tasks to remediate these contaminants are not easy and are expensive to carry out, due to the diverse nature of the contaminants found in the environment and to the very many geographical sites affected by chemical contamination.

Conventional approaches to the remediation of chemical contaminants using bio-sand, precipitation, reverse osmosis, adsorptive filtration through ion exchange resins, adsorption by active alumina and iron oxide can only remove certain compounds which are typically heavy metals.^3^ Besides that, there are other major problems found with the use of these methods which are incomplete precipitation of the contaminant and the formation of large volumes of sludge that is known to be difficult to be filtered and disposed of.^4^ Among the different treatment methods used for chemical decontamination, the adsorption method is considered the simplest and most convenient approach.^5^ Adsorption is a surface process that involves the accumulation of a gas or liquid onto a solid phase which is called the adsorbent. Activated carbon and zeolites have been used and are the most popular adsorbents used for the removal of chemical contaminants from waste effluents as well as from the atmosphere.^2,3,6,7^ However, these materials have restricted applicability because of their non-renewable physical form and high cost especially for activated carbons that suffer from material losses during regeneration processes.^8^

Nowadays, the use of lignocellulosic biomass as an alternative material to replace other synthetic materials, for environmental remediation has attracted much interest.^3,9^ Malaysia annually generates 6.93 million tons of oil palm biomass (dry basis) which then provides a potential bioresource for conversion into value-added products such as chemical feedstocks, biosugars, biofuels, biophenol, bioplastic, cellulose, nanocellulose and for use in composite production.^10–13^ Lignocellulosic biomass is an amazing raw material with abundant availability, resource that is renewable, sustainable in the long term, 100% biodegradable and has no known side effects to the environment. However, untreated biomass is generally not functional due to the presence of lignin and hemicellulose in it. In order to make this biomass a useful resource, the cellulose within it can be isolated and processed at a nano-scale level which then improves the usefulness of this material as a new generation of sustainable adsorbents. When the particle size of the extracted cellulose is minimized to nanoscale, the high adsorption specific area of the resulting material results in an enhanced adsorption capacity. Interesting, Jin *et al.* (2011)^14^ reported that nanocellulose was capable of adsorbing 40 times more than its own weight.

Thanks to its broad range of possible functionalization, reactivity, processability, biodegradability and reversibility, nanocellulose has emerged as a new class of biobased adsorbent with promising applications in environmental remediation against a large family of contaminants.^3^ The functionalization of nanocellulose is key to its success as an adsorbent especially together with its excellent adsorption capacity. Several interesting findings from the functionalization of nanocellulose have been discovered by scientists. When compared to inorganic nanoparticle adsorbents such as carbon nanotubes, titanium dioxide (TiO_2_), cerium oxide (CeO_2_), zinc oxide (ZnO), iron oxide, zero-valent metals, fullerenes, nanocellulose was found to exhibit better or comparable adsorbent capacity. Besides that, the use of nanocellulose eliminates the safety concerns generally encountered when using mineral and carbon nanoparticles.^15^

This review highlights recent progress and the trends related to the application of nanocellulose as an adsorbent for chemical contaminants. The detailed characteristics of nanocellulose which form the necessary requirements for an adsorbent shall be looked at. This review also includes a discussion on the wide possibilities for the functionalization of nanocellulose. Following which, the capability of nanocellulose as an adsorbent for heavy metal, chemical dye and organic oil contaminants as has been reported in relevant recent literature shall be looked at. For comparison purposes, the adsorption capacity of several other available adsorbents shall also be used as a comparison.

## 2. Chemical contaminants

As mentioned earlier, there are many chemical contaminants that can affect the environment and our health. Most chemical contaminants enter the environment from industrial activities and from human habitation sources. These sources of contamination can be classified as either point or non-point sources.^16^ Point sources are defined as identifiable points or places that are easily located. Whereas, non-point sources are those where it is difficult to identify the exact origin of the contamination.

The World Health Organization (WHO) through its International Programme on Chemical Safety has identified ten chemicals (this list includes groups of chemicals) of major public health concern.^17^ These chemicals are air pollution, arsenic (As), asbestos (silicate minerals), benzene (C_6_H_6_), cadmium (Cd), dioxin and dioxin-like substances, inadequate or excess fluoride (F^−^), lead (Pb), mercury (Hg) and highly hazardous pesticides.

**Fig. 1:**
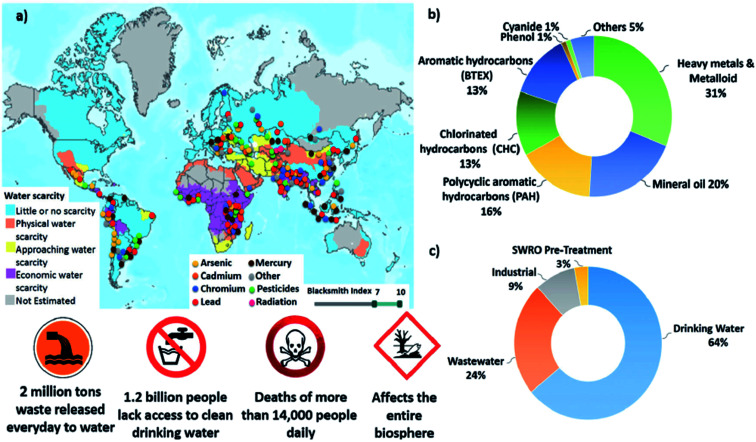
Summary of worldwide global water contamination spread in numbers. (a) Superimposed geographical distribution of water scarcity and of contamination by the type of major contaminants, including heavy metals, pesticides and radionuclides. (b) Normalized composition of water contaminants in treated and recycled water streams. (c) Normalized volume of treated water by field of use. Reproduced from ref. 2 with permission from The Royal Society of Chemistry.

Water rich areas such as rivers and lakes, soil moisture and groundwater in aquifers and the oceans are the locations known to have the highest load of chemical contaminants. Moreover, many other points of contamination from both land and atmospheric sources are also linked to water sources. Liquid waste containing toxic chemicals on terrestrial surfaces of land masses can seep slowly into the soil and eventually percolate into groundwater leading to its contamination. Besides that, rainwater which washes toxic contaminants floating in the air also adds to the problem when the rain falls onto land masses and eventually enters the groundwater sources or flows into rivers and lakes or even the ocean. The environmental water cycle connects all these different environments and causes environmental contamination to be pervasive and wide-spread, occasionally even across continental land masses. Bolisettyl *et al.* (2019)^2^ critically reviewed worldwide global water contamination and their findings are as shown in Fig. 1. According to them, heavy metals are the largest chemical contaminants in wastewater. There are about 14 000 people who die every day because of this water contamination.

Besides heavy metals, dyes, and organic oils, there are several other chemical contaminants that can also cause intoxication to living organisms involving both fauna and microorganisms. These contaminants include various acids, plant nutrient chemicals, organophosphorus and organochlorine compounds. However, the volume of these contaminants in the environment are relatively less as compared to heavy metals and organic dyes as shown in Fig. 1. Table 1 summarizes all types of chemical contaminants, their origin and health effects after exposure.

**Table 1:** Health effects of exposure to several other chemical contaminants

Chemical contaminants	Origin	Health effect
Heavy metals	Metal-bearing rocks, volcanic eruptions, mining activities, various industrial and agricultural activities	The toxic levels of heavy metals in our water resources are known to trigger cellular damage in body tissues such as the brain, lungs, kidneys, liver and blood. Furthermore, chronic exposure to heavy metal contamination can cause a variety of health problems and diseases and manifest as cancers, Parkinson's disease, muscular dystrophies, kidney stones, bone diseases, osteoporosis. and Alzheimer's disease, among others^18–22^
Dyes	Waste material or from the by-products of the agricultural, forestry, food, petroleum products and beverage industries^23^	The use of synthetic dyes has created much waste that can then in turn seriously affect the Biochemical Oxygen Demand (BOD), Chemical Oxygen Demand (COD), pH value and total suspended-solid (TSS) of environmental water^24^
		Dyes waste are toxic and usually also carcinogenic especially when it has accumulated within the human body and would by then cause serious health problems
Organic oils	Spillage of organic oils such as silicon, vacuum pump, paraffin, diesel, petroleum ether, silicon, canola, pump and motor	It has caused serious contamination and threatens the environment, aquatic organisms and has also already harmed human health^25,26^
Acids	Acid rain, mine drainage, industrial waste discharge, acidified soils	Acidification of ground water can lead to the death of numerous living organisms and microbes
Phenols	Mainly from industrial effluent releases into water ways, the atmosphere and onto land	Phenols can cause severe health effects such as cardiovascular disease, gastrointestinal damage. Long term exposure is known to cause damage to the heart, kidneys, liver, skin and lungs
Plant nutrient chemicals	Nitrates from agricultural fertilizers in sewage effluents and field water run off	Causes algae blooming which can lead to the death of submerged vegetation
Organophosphorus	Found in sewage effluents as phosphate compounds originating from herbicides and pesticides	Organophosphorus poisonings affecting many organ systems through muscarinic and nicotinic effects and causing central nervous system debility^27,28^
Chlorinated hydrocarbons (organochlorine compounds)	From agricultural runoff, crop spraying, waste incineration, and toxic dumps	They act as endocrine disrupting chemicals (EDCs) by interfering with the function of the endocrine system.^29^ Direct or indirect exposure leads to neuromuscular disorders and stimulation of steroid metabolism.^30^ Biomagnification is also know to occur, where top carnivores, including humans, can be affected

## 3. Current approaches to eliminate chemical contaminants

There are several conventional water treatment methods such as bio-sand, precipitation, reverse osmosis, biodegradation, photocatalysis, adsorptive filtration through ion exchange resins, active alumina and iron oxide can be used for removal of contaminants.^31–42^ However, some of these techniques can only remove certain chemical contaminants. Although adsorption–filtration is often the method of choice, chemical oxidation, membrane separation, liquid extraction, electrolytic treatment, electro-coagulation, electro-dialysis, electrochemical treatment, membrane technologies and adsorption onto activated carbons have also been used for many years to purify potable water.^43^ The major problem using these methods are incomplete precipitation and the formation of large volumes of sludge that then are difficult to filter out for disposal. Another restriction to their widespread application is the high cost especially for activated carbons that themselves suffer from material losses during regeneration after use.

Adsorption has been recognized as an attractive approach for the removal of chemical contaminants due to its simple method of application, its being free from producing of bi-products and are cheaper as compared to other approaches.^44–46^ Adsorption allows contaminant molecules to attach to the surface of an adsorbent by physical forces such as van der Waals forces and electrostatic attraction or through covalent bonding between oppositely charged adsorbate molecules and the adsorbent surface.^47^ However, adsorbent quality degrades after multiple cycles of use and the adsorption column needs to be maintained and cleaned periodically.^2^ However, not all adsorption-based techniques can operate in a broad range of environmentally relevant pH conditions. Besides pH, other condition parameters such as contact time, the concentration of the contaminant and operating temperature also play a critical role on adsorption capacity.^48^

Generally, ideal materials for the adsorption of contaminants should meet several requirements: (1) be inexpensive, (2) have good mechanical and structural integrity to withstand water flow over a long period, (3) show high adsorption capacity with a high rate, (4) have a large specific surface area and (5) possess regeneration aptitude using cost-effective approaches.^3^ Examples of the most commonly used materials as potential adsorbents are shown in Table 2 in accordance with their class.

**Table 2:** Several classes of frequently used adsorbent materials

Type of adsorbent	Materials
Inorganic	- Zeolites
	- Clays
	- Silica gel
	- Activated alumina
	- Pillared clays
	- Metal oxides and hydroxides
Polymeric	- Membranes
	- Ion exchange resins
	- Molecularly imprinted polymers
Carbon	- Activated carbon
	- Fullerenes
	- Heterofullerenes
	- Mesocarbon
	- Molecular carbon sieves
	- Carbon nanotubes
Biobased	- Cellulose from various plants
	- Nanocellulose

Activated carbon has been used as the most popular adsorbent for the removal of a myriad of contaminants ranging from liquid effluents to those airborne in the atmosphere.^3^ Activated carbon exists in different physical forms, including: (1) granular activated carbon, (2) powdered activated carbon, (3) activated carbon fibres and (4) activated carbon cloths. The most important characteristic of activated carbon it its pore size distribution, surface chemistry and mineral matter content. Functionalization of this material has also been extensively being studied. Most of the functionalized adsorbents developed have an improved adsorption capacity towards contaminants as compared to their unfunctionalized counterparts. The effectiveness of several examples of adsorbents other than nanocellulose towards heavy metal, dye and organic oil removal are shown in Table 3, 4 and 5, respectively.

**Table 3:** Adsorption of heavy metals by several adsorbents

Adsorbent	Contaminants	Adsorption capacity	Reference
Activated carbon	Cd(ii)	31 mg g^−1^	49
Activated carbon	Zn(ii)	29 mg g^−1^	49
Palygorskite clay	Pb(ii)	62 mg g^−1^	50
Palygorskite clay	Ni(ii)	33 mg g^−1^	50
Palygorskite clay	Cr(vi)	59 mg g^−1^	50
Palygorskite clay	Cu(ii)	31 mg g^−1^	50
Thiol-functionalized activated carbon	Cu(ii)	88 mg g^−1^	51
Thiol-functionalized activated carbon	Pb(ii)	238 mg g^−1^	51
Thiol-functionalized activated carbon	Cd(ii)	96 mg g^−1^	51
Thiol-functionalized activated carbon	Ni(ii)	52 mg g^−1^	51
Amino-functionalized activated carbon	Cd(ii)	79 mg g^−1^	52
Amino-functionalized activated carbon	Pb(ii)	142 mg g^−1^	52
Thiol-functionalized activated carbon	Cd(ii)	130 mg g^−1^	52
Thiol-functionalized activated carbon	Pb(ii)	232 mg g^−1^	52

**Table 4:** Adsorption of dyes by several adsorbents

Adsorbent	Contaminants	Adsorption capacity	Reference
Activated carbon	Acid blue	203 mg g^−1^	53
Activated carbon	Methylene blue	252 mg g^−1^	54
Activated carbon	Methylene blue	180 mg g^−1^	55
Activated carbon	Methylene blue	91 mg g^−1^	56
Activated carbon	Reactive blue 2	0.27 mmol g^−1^	57
Activated carbon	Reactive blue 4	0.24 mmol g^−1^	57
Activated carbon	Reactive yellow 2	0.11 mmol g^−1^	57
Silica	Acid blue 28	333 g kg^−1^	58
Silica	Acid blue 113	769 g kg^−1^	58
Zeolite	Everzol Red 3BS	111 g kg^−1^	59
Zeolite	Everzol Black B	61 g kg^−1^	59
Chitosan grafted with amide	Remazol Yellow Gelb 3RS (reactive dye)	1211 mg g^−1^	60
Chitosan grafted with carboxy	Basic dye (Basic Yellow 37)	595 mg g^−1^	60
Cross-linked chitosan (CCS)/bentonite (BT) composite	Azo dye (Amido Black 10B)	324 mg g^−1^	61

**Table 5:** Adsorption of organic oils by several adsorbents

Adsorbent	Contaminants	Adsorption capacity	Reference
Polyurethane	Light crude oil	19 g g^−1^	62
Polyurethane	Diesel	47 g g^−1^	63
Polyurethane	Kerosene	41 g g^−1^	63
Nanoclay-polyurethane	Light crude oil	22 g g^−1^	62
Polypropylene	Crude oil	7–15 g g^−1^	64
Magnetic carbon	Engine oil	10 g g^−1^	65
Magnetic carbon	Chloroethane	11 g g^−1^	65
Magnetic carbon	Corn oil	10 g g^−1^	65
Felt (NOAF-1) (commercial oil adsorbent)	Crude oil	8 g g^−1^	66
Expanded perlite	Heavy crude	3 g g^−1^	67
Expanded perlite	Light cycle	4 g g^−1^	67
Hydrophobic nano-silica	Diesel	14 g g^−1^	66
Macroporousorganogel	Gasoline	15 g g^−1^	68
Macroporousorganogel	Toluene	21 g g^−1^	68
Macroporousorganogel	Crude	18 g g^−1^	68
Cotton grass fiber	Diesel	20 g g^−1^	62
Cotton grass fiber	Gasoline	19 g g^−1^	62
Ferric oxide nanoparticles doped carbon nanotubes	Gasoline oil	7 g g^−1^	69
Lauric acid modified oil palm leaves	Crude oil	1 g g^−1^	70
Ceramic matrix composite/Na^+^– montmorillonite	Waste pump oil	20 g g^−1^	71
Polystyrene/Fe_3_O_4_/graphene aerogel	Crude oil	40 g g^−1^	72
ZnFe_2_O_4_ porous silicone	Dichloromethane	11 g g^−1^	73
ZnFe_2_O_4_ porous silicone	Toluene	9 g g^−1^	73
Fe_2_O_3_-PAMAMOS	Engine oil	23 g g^−1^	74

## 4. Characteristics of nanocellulose

Nanocellulose can be defined as a cellulose having a dimensions of 100 nanometers (nm) or less.^12,75–77^ Before the production of nanocellulose, cellulose can be extracted from a broad range of plants, animals, and bacteria. A wide variety of plant materials such as wood, oil palm biomass, bamboo, rice husk, sisal, hemp, flax, kenaf, and coconut husk have been studied as a source of cellulose for nanocellulose production.^9,77–80^ Tunicates are a type of marine invertebrate which is a member of the subphylum Tunicata which contains cellulose.^80^ Otsuka *et al.* (2017)^81^ successfully produced nanocellulose from tunicates with properties comparable to plant-based nanocellulose. Nanocellulose is classified as variously as cellulose nanofiber (CNF), cellulose nanocrystal (CNC) and bacterial nanocellulose (BNC).^82–84^ Different approaches have been used to extract these three types of nanocellulose, resulting in different properties of their crystallinity, surface chemistry and mechanical properties.^84^ Interest on the applications of nanocellulose are increasing and not only focus on its adsorbent properties. It has been applied for several other applications in the fields of material science, biomedical engineering, cosmetics, pharmaceuticals, textiles, electronics, foods and packaging.^85–94^

The effect of morphology of nanocellulose on the removal of contaminants is important to be considered in order to produce an effective adsorbent. Nanocellulose fibrils are linear and aggregation usually occurs *via* both intra- and intermolecular hydrogen bonds. Usually, nanocellulose has a strong affinity to itself and toward materials containing hydroxyls groups. As a result, nanocellulose is known to be easily agglomerated if the proper drying technique does not apply. This phenomenon is known as “hornification”.^95^ The mechanism of hornification is described as the formation of coalesced microfibrils by irreversible hydrogen bonds. The aggregated morphology of nanocellulose decreases the specific surface area of the fiber, thus it will negatively affect the adsorption capacity.

To overcome this problem, nanocellulose aerogels have become increasingly interesting to researchers when used as an adsorbent. The term “aerogel” is commonly used interchangeably to describe nanocellulose-based porous materials.^96^ Aerogels are extremely porous materials that have very low densities (0.01–0.4 g cm^−3^), and a large total specific surface area (30–600 m^2^ g^−1^). Aerogel was discovered by Kistler (1931). They are prepared by sublimating a liquid component through lyophilization (freeze-drying) or by critical point drying to remove the solvent to become a high porous, lightweight and networked material.^97–99^ Nanocellulose aerogels can be produced in three steps which are dispersing or dissolving cellulose derivatives, then a sol–gel process takes place to form a cellulose gel and then this cellulose gel undergoes a drying process without disturbing its 3D porous structure.^100^ As compared to inorganic aerogels, nanocellulose aerogels have several advantages as follow:^3^

(a) Nanocellulose aerogel is considered more environmentally friendly than the other types of aerogel since they can be synthesized from renewable resources. Several research have managed to eliminate the use of harmful chemicals in their processing methods.

(b) Nanocellulose aerogels can provide very large specific surface, depending on the drying method and width of the cellulose fibrils.

(c) Nanocellulose aerogels are highly flexible and good mechanical performance as compared to other type of aerogels.

(d) The density and porosity of the nanocellulose aerogels can be theoretically predicted.

### 4.1 Attributes of nanocellulose as an adsorbent

**Fig. 2:**
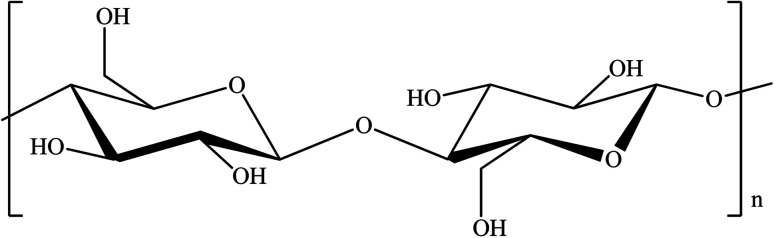
Chemical structure of cellulose.

As mentioned above, nanocellulose is known to be a versatile material because of its interesting properties. The ability to functionalize the surface chemistry of nanocellulose makes it an excellent material as an adsorbent of chemical contaminants. The hydroxyl groups, –OH on the cellulose backbone of nanocellulose enables facile functionalization of nanocellulose that introduces the desired functionality and produces highly effective flocculants.^101^Fig. 2 shows the chemical structure of cellulose where it contains an abundance of hydroxyl groups.

Other than that, interesting characteristics of nanocellulose include an extremely high specific area, capability for repeated adsorption and desorption, high porosity with excellent pore interconnectivity, low weight, high biodegradability, and rod- or ribbon-like structure.^75,78,102^Table 6 summarizes the importance of these special characteristics of nanocellulose in its application as an adsorbent.

**Table 6:** Several properties of nanocellulose related to adsorbent properties

Property	Advantages	Reference
Surface functionalization	Able to be surface functionalized through oxidation, esterification, etherification, silylation, addition and grafting. This causes an increase in adsorption capacity. Example of possible surface functionalization aiming to enhance the adsorption capacity are discussed in the next section	103
Reusable/desorption	Ability to be reused many times to adsorb and desorb contaminants. Nanocellulose only requires a simple method of regeneration without negative effects on its adsorption capacity	3, 104 and 105
Renewable	Cost effective compared to activated carbon. Can be utilized form several biomass	106
Biodegradability	It is biodegradable. Thus, it is not harmful to the environment	3
High specific surface area	It provides large number of active sites for functionalization. This will also increase the adsorption capacity	107
High mechanical properties	The high stiffness and cohesion of nanocellulose improves the mechanical properties of the adsorbent. This offers the possibility for regeneration as an adsorbent	108
Good surface tension properties	Favoring the wetting of nanocellulose by water	109
Stable in water	High hydrophilicity of nanocellulose can reduce bio- and organic-fouling. The high crystallinity of nanocellulose, makes the adsorbent resistant to biological and chemical corrosion in water	110

## 5. Functionalization of nanocellulose

**Fig. 3:**
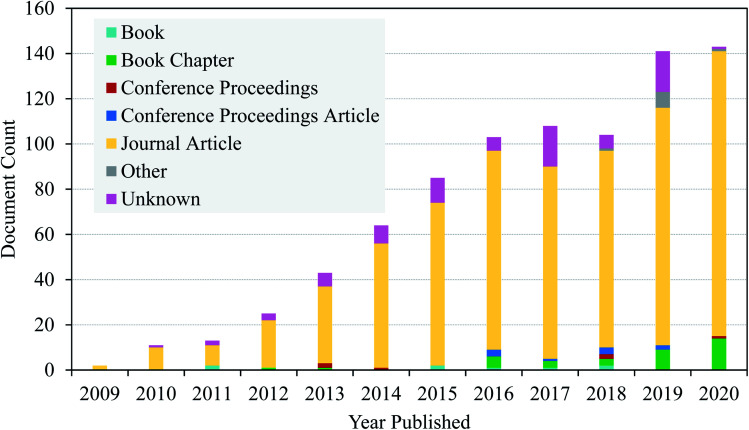
A chart of published manuscripts focussing on the functionalization of nanocellulose.

As discussed above, nanocellulose in its native form has limitations in its application as an adsorbent. Therefore, various functionalization approaches have been applied to increase its surface polarity and hydrophilicity.^111^ Modification by surface functionalization is a key step in promoting its adsorption towards a specific class of contaminant and thus enhances its adsorption capacity.^112,113^ This can be done using different strategies of surface functionalization which typically involve the chemistry of hydroxyl function.^103^ Besides that, as is seen in Fig. 3, a search was done on lens.org using the keyword ‘functionalization of nanocellulose’ and it was found that manuscripts focusing on the functionalization of nanocellulose has increased in recent years. Thus, this shows that research on the functionalization of nanocellulose continues to gain much interest among scientist in this decade. There are several applications of functionalized nanocellulose such as environmental remediation, biomedical, packaging, sensor technology, papermaking, and automotive.

Generally, modification of adsorbents has several targets depending on types of contaminant. The mechanism of action between the adsorbent and contaminant need to be considered. For heavy metals and dyes, the charged surface of adsorbents is preferable. Normally, the negatively charged adsorbent will effectively adsorb the positively charged heavy metals and dyes. Meanwhile, considering the hydrophobic and oleophilic characteristic of oils, the hydrophobic groups with a low surface energy are commonly introduced to nanocellulose by using physical or chemical methods to improve their oil adsorption capacity. On the other hand, the functionalization process must be environmentally friendly. The complex processes, high cost, large amount of chemicals and high energy usage of the functionalization process need to be avoided. This is to ensure there is no consequence generation of chemical contaminants are produced from the process. Moreover, the degradation issue of the functionalized nanocellulose also need to be considered. The adsorbents which can be degraded naturally are preferable. The desorption capability of the developed adsorbent is one of the vital aspects. Usually, the functionalized nanocellulose must have the ability to reabsorb the contaminants for several cycles without lower its performance. The performance of adsorbent also can be improved by introducing the magnetic compound to its chemical structure. The super-magnetized nanocellulose can be easily collected after the remediation process.

Several functionalization approaches have often been applied to nanocellulose such as cationic interaction, silylation, acetylation, coupling with isocyanate derivates, and succinic anhydride. Table 7 illustrates examples of functionalization of nanocellulose for chemical contaminant remediation as discussed in this review. The process and mechanism involved were also highlighted in this table. Besides that, any issue related to the environmental concerns of the functionalization process were also highlighted in this table. Details on the adsorption capacity and its desorption study of these functionalized nanocellulose towards heavy metals, dyes and organic oils will be discussed in the next section.

**Table 7:** Examples of modified nanocellulose used for chemical contaminant remediation

Developed adsorbent	Modification process and mechanism of action	Environmental side effect of the modification process	Reference
**Contaminant: heavy metal**			
Carboxyl functionalized nanocellulose (In-situ TEMPO functionalization)	- Two layers of nanocellulose (CNF and CNC) membranes were developed	- There is no harsh chemicals and no complex processes involved in this functionalization	114 and 115
	- The support layer of CNF is to increase the water permeability due to the porous network structure	- However, TEMPO is a toxic chemical to aquatic life that cannot be released into waste effluent after the oxidation, as it can accumulate in the environment^114^	
	- The functional layer of CNC was treated using 2,2,6,6-tetramethylpiperidine-1-oxyl radical (TEMPO)–NaBr–NaClO system *via in situ* functionalization aimed to attach carboxyl functional group		
	- The carboxylated nanocellulose has large numbers of carboxylic acid functionalities that are effective for binding metal ions		
Nanocellulose-based polyethyleneimine (amino functionalized nanocellulose)	- Amino functionalized CNF was prepared *via* self-assembly of TEMPO oxidized CNF crosslinked trimethylolpropane-tris-(2-methyl-1-aziridine)propionate (TMPTAP) and polyethyleneimine (PEI)	- In the functionalization process, the cross-linking approach wherein aziridine reacted with carboxyl groups *via* the spontaneous ring-opening reaction in liquid at room temperature is considered as a facile, cheap and efficient process	104
	- The developed adsorbent showed a 3D multi-wall perforated cellular structure with plentiful amino groups and oxygen-containing groups	However, TEMPO is a toxic chemical to aquatic life	
	- The presence of the amino group plays an important role in concordance with Cu(ii) in the adsorption process		
	- The developed adsorbent can anchor Cu(ii) ions and form tetradentate- or bidentate-coordination cyclic chelate		
Nanocellulose functionalized with polyethyleneimine and glutaraldehyde with the presence of “bridge effect” of iron ions	- The functionalization was carried out by the carboxylation of nanocellulose with TEMPO and cross-linked with PEI *via* peptidic coupling. Glutaraldehyde is added as cross-linker agent to strengthen the cross-linking between nanocellulose and PEI	- The residual chemicals can be produced through this functionalization process which might be harmful to the environmental	116
	- Similar as above, the presence of TEMPO and PEI is to increase the adsorption capacity towards heavy metals		
	- The presence of Fe ions successfully connects the two dispersed polymers together, inducing large numbers of O–Fe–O bonds and, providing more adsorption active sites for the removal of heavy metals		
Magnetic carboxylated nanocellulose	- Magnetic separation has been proven to be a convenient approach for the removal of heavy metals from water	- Fe_3_O_4_ nanoparticles are widely used in magnetic materials electrically conductive materials and biomedicine because of their excellent features: simple preparation, low cost, environmental compatibility and good magnetic properties	117
	- Same as above, the carboxylated nanocellulose has large numbers of carboxylic acid functionalities that are effective for binding metal ions	- The functionalization process also does not require the use of harmful solvents	
	- The carboxylated nanocellulose was treated with Fe_3_O_4_ nanoparticles *via* co-precipitation method		
	- The presence of carboxyl and hydroxyl groups on the surface of the developed adsorbent are important sites for adsorption to occur		
Thiourea-functionalized magnetic ZnO/nanocellulose	- Similar as above, the presence of magnetic Fe_3_O_4_ and ZnO will lead to an effective adsorbent to remove of heavy metals	-This functionalization process requires simple preparation, low cost and environmental compatibility	118
	- Thiourea-functionalized magnetic nanocellulose was prepared using a facile chemical co-precipitation method	- No harmful solvents were used in this functionalization. Therefore, no secondary pollution can be produced	
	- The presence of OH or NH_2_ and S groups dominates the adsorption of heavy metals		
	- The ion exchange mechanism plays an important role in improving the adsorption process. N-Metal ion, S-metal ion and O-metal ion possess a lone pair which donates their electron to form a complex with metal ions through the sharing of the electron pair		
Chitosan/phosphorylated nanocellulose	- Similar to nanocellulose, chitosan is well known for its ability to remove heavy metals	- This functionalization procedure is safe because chitosan and phosphorylated nanocellulose are renewable, biodegradable, cheap, and easily available	119
	- Phosphorylated nanocellulose also been proven to have high adsorption capacity towards heavy metals	- The electrospinning process is a simple manufacturing technology and no secondary pollution is produced	
	- Phosphorylated nanocellulose was introduced to chitosan matrix *via* electrospinning method. Poly (ethylene oxide) PEO is used as copolymer agent to decrease chitosan viscosity and increase electrospinnability		
Nanobentonite incorporated nanocellulose/chitosan	- Nanobentonite is known as very inexpensive strong adsorbent due to its incredible density and availability. The effective adsorption mechanism in nanobentonite is due to the Si(iv) and Al(iii) isomorphous replacement in the silica layer resulting in constant negative charges on its surface	- Bentonite can be obtained naturally. Moreover, no hazardous chemicals are used during the sonication process	120
	- Nanocellulose, nanobentonite and carboxymethyl chitosan were added gradually and sonicated in ice bath to form dialdehyde nanocellulose-carboxymethyl chitosan		
Nanocellulose functionalized with activated carbon	- Activated carbon also known as an effective adsorbent	- The functionalization method reported is done by using homogenization. There are no other chemicals used throughout the process. Therefore, this approach does not produce the secondary pollution	121
	- The nanocellulose was functionalized with activated carbon by using simple homogenization process	- However, the production of activated carbon itself can be harmful to the environment	
Nanocellulose acetate functionalized with hydroxyapatite	- The nano-hydroxyapatite is known to have high adsorption capacity for metal ions, low water solubility, availability, low cost and high stability under oxidizing and reducing conditions^33^	- The functionalization method requires the use of several solvents such as dimethylformamide, which might be harmful to the environment	122
	- The nano-hydroxyapatite was synthesized by using wet chemical precipitation method. Meanwhile the functionalization of CNF is done *via* electrospinning process		
	- The adsorption mechanism on the functionalized CNF is established *via* ion exchange and surface complexation		

**Contaminant: dyes**			
Nanocellulose/carboxymethylated chitosan	- The carboxymethylated chitosan is used to increase the carboxyl group content	- There is no harmful chemical used in the development of carboxymethylated chitosan and the functionalization method	123
	- This adsorbent was synthesized from crosslinking bifunctional nanocellulose and carboxymethylated chitosan through a Schiff base reaction	- Thus, this can avoid the formation of secondary pollution	
	- This developed adsorbent is a negatively charged. Thus, it capable to adsorb the positively charged dyes		
	- The adsorption mechanism between this developed nanocellulose and methylene blue is by electrostatic attraction between the acidic groups in the anionic nanocellulose and the dye		
Electrosterically stabilized nanocellulose	- Electrosterically stabilized CNC (ECNC) was prepared through a two-step oxidation by periodate and chlorite	- This functionalization technique is considered as environmentally friendly and cost-effective procedure	124
	- The developed adsorbent has high negative charge density. Thus, it can have high adsorption capacity towards positively charged dyes		
	- The adsorption of cationic dye is based on an ion-exchange mechanism and influenced by the presence of other ions		
Nanocellulose functionalized with polyvinylamine (PVAm)	- PVAm is known to have high content of amine groups	- No harmful solvents were used in the functionalization process. The unreacted PVAm and dialdehyde nanocellulose can be collected using distilled H_2_O	125
	- Similar to heavy metals, amino functionalized nanocellulose showed an outstanding adsorption capability for anionic dyes, since amino groups are easily protonated under acidic conditions		
	- Two steps were involved where dialdehyde nanocellulose was produced first using sodium periodate, then the dialdehyde nanocellulose acts as a crosslinker in reaction with PVAm to generate an adsorbent with a high content of amine groups		
	- Various functional groups on the nanocellulose-PVAm such as hydroxyl, carboxyl and amines influenced electrostatic attraction between the adsorbent and target dyes		
Nanocellulose functionalized with polypyrrole	- Polypyrrole has a capability to adsorb several heavy metals and dyes	- Polypyrrole is an organic polymer formed from polymerisation of pyrrole ring, which has good stability, low cost and eco-friendly	126
	- The sonicated nanocellulose was mixed with polypyrrole. The functionalization method was carried out by lyophilisation. The addition of ammonium persulphate provides free radicals and helps in the cross-linking between nanocellulose and polypyrrole	-The functionalization process by sonication also does not require the use of harmful chemicals	
	- The adsorption mechanism of dyes by the developed adsorbent is an endothermic, spontaneous, and entropy-driven process		
Polydopamine/nanocellulose	- Polydopamine (PDA) is rich in catechol and amine groups, which facilitate covalent conjugation or other noncovalent interactions with organic and inorganic materials	- This functionalization process is considered as low cost, scalable, environmentally friendly, and reusable	127
	- Polydopamine/nanocellulose was synthesized by *in situ* incorporation of PDA particles into BNC matrix during its bacteria-mediated growth	-However, the synthesize method of polydopamine requires the use of ammonia which is toxic to human	
Meldrum's acid modified nanocellulose	- Meldrum's acid is known as an esterification agent to enhance the adsorption toward positively charged crystal violet dyes	- It is a solvent-free surface treatment which will not cause harmful to the environment	128
	- It is a new treatment to modify nanocellulose using solvent free technology		
	- Meldrum's acid was functionalized with nanocellulose *via* esterification to attach COOH functional group using nonsolvent assisted method. The modified nanocellulose is impregnated onto polyvinylidene fluoride (PVDF) membrane by applying pressure before heated for 15 minutes to immobilize the CNF network to cross-linking with the polymer membrane		
Nanocellulose functionalized with magnetic nanoparticles (Fe_3_O_4_)	- The use of magnetic nanoparticle functionalized nanocellulose also been tested for dyes adsorption	- The fabrication of this magnetic adsorbent requires a simple method (vacuum filtration) which is considered as inexpensive and scalable	129
	- Similar as above, the function of magnetic nanoparticle is to improve the magnetic separation	- The developed adsorbent is environmentally friendly and offer the advantages of cost-effectiveness and easily degraded over other metal-based catalysts	
	-The magnetic nanoparticles were grafted to the surface of nanocellulose through *in situ* hydrolysis of metal precursors at room temperature		
Carbon-phosphorus-titanium (nanocellulose used as carbon precursor)	- Titanium dioxide is applied due to a high photocatalysis activity, low cost, relative low toxicity and good chemical and thermal stability	- Titanium dioxide is low toxic. However the functionalization requires complex processes, high energy and time consuming	130
	- The developed adsorbent is used to adsorbed orange-dye and degrade it by photocatalysis		
	- Carbon–phosphorus–titanium composites were synthesized by Ti-impregnation and carbonization of cellulose		

**Contaminant: organic oils**			
Nanocellulose functionalized with titanium dioxide	- The titanium oxide is known as hydrophobic and oleophilic	- The synthesis method of titanium dioxide and functionalization process is not environmentally hazardous	131
	- This functionalization is to increase the hydrophobicity and oleophilic coating on the nanocellulose's surface. Consequently, this can increase its ability to adsorb nonpolar liquids and oils		
	- The titanium oxide was coated to the nanocellulose using atomic layer deposition		
Nanocellulose based-carbon	- The hydrophilicity properties of nanocellulose can be reduced through functionalization and conversion by pyrolysis into carbon nanocellulose	- However, this approach might not be considered as environmentally or economically desirable because it is obtained either by means of complex syntheses that require large amounts of chemical reagents, or through high-energy input processes	132
	- Nanocellulose and crosslinking agent were oven-heated to promote cross-linking in order to form a three-dimensional network. The nanocellulose was stabilized in air and carbonized in nitrogen using a tube furnace		
Magnetic/silanized ethyl nanocellulose	- Different from nanocellulose, the ethyl cellulose has a hydrophobic and oleophilic properties	- This functionalization technique is considered as environmentally friendly. No secondary pollution is generated throughout the process	133
	- Silanization is used to increase the hydrophobicity of the nanocellulose		
	-As mentioned before, the used of Fe_3_O_4_ nanoparticles is to improve the magnetic separation		
	- The superhydrophobicity and magnetism of the adsorbent was achieved by silanization the ethyl cellulose with hexadecyltrimethoxysilane and mixing with Fe_3_O_4_ nanoparticles		
3D skeleton nanocellulose	- The superhydrophobicity and superoleophilicity of the nanocellulose surfaces can be obtained by constructing a rough nanostructure and introducing a low surface energy substrate	- This modification approach does not require used of hazardous chemicals. It does not produce the secondary pollution	134
	- The method of preparation involves two steps. Firstly, the nanocellulose aerogel was synthesized. The resulting aerogel has perfect 3D skeleton and interconnected pores similar to the honeycomb. Secondly, the nanocellulose aerogel was directly constructed by a simple plasma irradiation		
Nanocellulose functionalized with stearoyl chloride	- The stearoyl chloride has a superhydrophobicity and superoleophilicity characteristics. Thus, it can adsorb oils effectively	- This developed adsorbent is considered as 100% natural and safe to the environment. This is because, no silylation agents were used in the production method	135
	- The adsorbent was fabricated by hydrophobic treated the nanocellulose with stearoyl chloride		
Nanocellulose functionalized with oleic acid and nanomagnetite (Fe_3_O_4_)	- This functionalization allows to combine both hydrophobic and magnetic responsivity properties. This could improve the adsorption capacity towards organic oils and magnetic separation of the adsorbent	- Similar as above, Fe_3_O_4_ requires simple preparation, low cost, environmentally friendly	136
	- Nanocellulose, oleic acid and Fe_3_O_4_ were mixed with deionized H_2_O through mechanical mixing	-The functionalization approach also does not produce the secondary pollution	

## 6. Nanocellulose as adsorbent of heavy metals

**Fig. 4:**
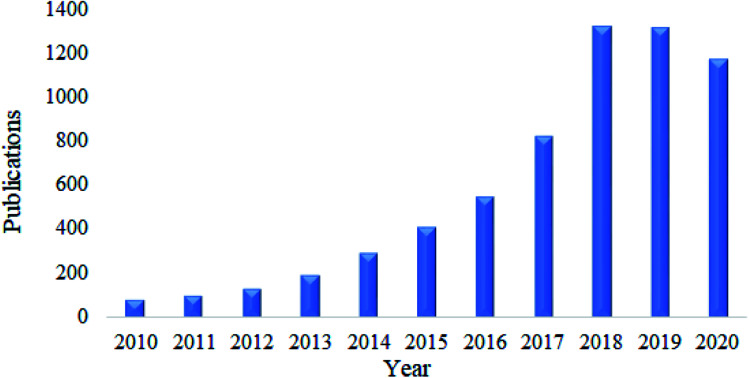
Total number of publications related to the nanocellulose as adsorbent of heavy metals.

Nanocellulose has been proven as an excellent adsorbent for the removal of heavy metals. The current trends on the publications related to this area are kept increasing extensively this past ten years. This can be seen by the survey done on Google Scholar using keyword “nanocellulose as adsorbent of heavy metal ions” as shown in Fig. 4. In this section, the adsorption capacity and desorption efficiency of several developed functionalized nanocellulose towards heavy metals as shown in Table 7 were reviewed. The adsorption capacity results reported here were compared with the currently available adsorbents for heavy metals as shown in Table 3.

Karim *et al.* (2017)^115^ developed the carboxyl functionalized nanocellulose for the removal of heavy metals (Ag(i), Cu(ii), Fe(ii) and Fe(iii)). Based on the results obtained, the adsorption capacity of Cu(ii) and Fe(ii)/Fe(iii) was increased up to 374 mg g^−1^ and 456 mg g^−1^, respectively as compared to unfunctionalized nanocellulose. This increment was 1.2–1.3-fold higher as compared to that of unfunctionalized CNF and CNC. Biodegradation studies of the developed adsorbent were carried out in wastewater and soil. There was no degradation observed for both unfunctionalized and functionalized nanocellulose at different pH condition of wastewater. For degradation in soil, a different pattern was observed. The unfunctionalized nanocellulose showed a 100% degradation after 15 days, meanwhile functionalized nanocellulose was degraded 87%.

Subsequently, Liuting *et al.* (2019)^104^ fabricated the amino functionalized 3D multi-wall perforated CNF-based polyethyleneimine (PEI) to remove Cu(ii) efficiently. The developed adsorbent showed a maximum adsorption capacity of 485.44 mg g^−1^ which was calculated using the Langmuir model. Desorption performance of functionalized CNF was determined using ethylenediaminetetraacetic acid (EDTA)-2Na. The ATR-FTIR curve of functionalized nanocellulose was restored completely after treatment with EDTA-2NA. This developed adsorbent was found to have great reversibility (4 cycles without significant degradation). The result of SEM-EDX mapping images also showed that structure morphology and nitrogen remained well dispersed after treatment with EDTA-2Na indicated chemical and structural stability.

The use of nanocellulose based-PEI has also been discovered by other researchers. Li *et al.* (2018)^137^ fabricated a functionalized CNF with PEI to remove Cu(ii) and Pb(ii). Based on thermodynamic and kinetic studies, it was shown that the pseudo-second-order and Langmuir model were the best fit. The maximum adsorption capacity of Cu(ii) and Pb(ii) reached 175.44 mg g^−1^ and 357.44 mg g^−1^, respectively, at pH 5.5 and its adsorption capacity was found to be retained for multiple usages. Besides that, the developed adsorbent could also be easily regenerated by EDTA and retain the adsorption capacity after repeatedly using. Recently, Chai *et al.* (2020)^116^ fabricated a functionalized CNC with PEI and glutaraldehyde in order to remove arsenic. The maximum adsorption capacity was 255.19 mg g^−1^. Based on the desorption study by using NaOH solution, the functionalized CNC displayed excellent performance even after eight cycles of desorption.

**Fig. 5:**
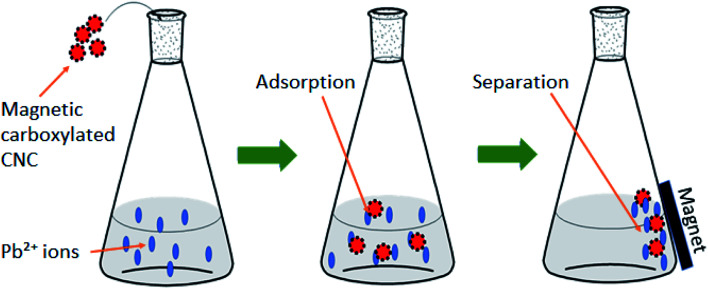
Adsorption process of Pb(ii) by magnetic carboxylated cellulose nanocrystals. Reproduced and adapted from ref. 117 with permission from Elsevier, copyright 2016.

Moreover, Lu *et al.* (2020)^117^ had developed a magnetic carboxylated CNC as a heavy metal adsorbent to remove Pb(ii). The adsorption capacity of Pb(ii) was 63.78 mg g^−1^ at 298.2 K with equilibrium adsorption being achieved at 240 min. Fig. 5 shows an example of the adsorption process of Pb(ii) by magnetic CNC. The developed adsorbent can be collected easily by using a magnetic force. The adsorbent could be regenerated through acid treatment and was successfully reused. The adsorption capacity was decreased as the cycles time increased. However, the performance of adsorbent still good with the capacity of 41.83 ± 0.79 mg g^−1^ and the removal ratio is more than 80%.

Using the same approach, Alipour *et al.* (2020)^118^ fabricated thiourea functionalized-magnetic ZnO/CNF as an adsorbent to remove Pb(ii). The results obtained showed that the developed adsorbent had great efficiency for removing Pb(ii) at pH 6.5, with a maximum adsorption capacity of 554.4 mg g^−1^ from a 60 mg L^−1^ of Pb(ii) concentration. The desorption studies were carried out by using HNO_3_ and ultrasonication. The result suggests that 15 s ultrasonic treatments after HNO_3_ could effectively regenerate the adsorbent with desorption efficiencies ranging from 96% to 100%. However, after the first cycle of desorption, the adsorption capacity of the adsorbent decreased 11% and after second cycle decreased 30%. Moreover, Saeid *et al.* (2018)^138^ also synthesized magnetic Fe_3_O_4_-nanocellulose to remove Hg(ii). The interaction of Hg(ii) and nanocellulose was studied using the Density Functional Theory (DFT) under various conditions. The results of the simulation showed good correlation with experimental data.

Using a combination of chitosan and phosphorylated nanocellulose is another alternative for fabricating efficient nanocellulose based adsorbents. Ricardo *et al.* (2019)^119^ produced chitosan/phosphorylated nanocellulose adsorbent to remove cadmium from aqueous solutions. The adsorption capacity was 62.3 mg g^−1^ at pH 5.5 with equilibrium adsorption reached within 120 min. The maximum adsorption capacity was 232.55 mg g^−1^ and the equilibrium was consistent with the Langmuir model. The high affinity of the amine and phosphate groups were the major determining factors for the excellent performance of the adsorbent. However, the desorption efficiency of the developed adsorbent was not reported in this study.

Shahnaz *et al.* (2020)^120^ fabricated nanobentonite incorporated nanocellulose/chitosan for the removal of cadmium (Cd), cobalt (Co) and Cu(ii) and used Response Surface Methodology (RSM) software to optimize the process variables. It was found that the adsorption capacity of Cr, Co and Cu were 2749.685, 916.65 and 1937.49 mg g^−1^, respectively. The desorption efficiency of the developed adsorbent was not reported.

Functionalization of nanocellulose using activated carbon was discovered by Athanasia *et al.* (2020).^121^ The CNC was synthesized from the empty oil palm fruit bunches before being functionalized with activated carbon. The developed adsorbent revealed great performance by achieving the removal of Pb(ii) at a maximum adsorption capacity of 24.94 mg g^−1^. The desorption study of the developed adsorbent was carried out by applying 1 M HCl solution followed by neutralization with 1 M sodium hydroxide and distilled water. The regenerated of the adsorbent can be re-used until three cycles. However, the adsorption capacity of second and third cycles was decreased to 9.42 mg g^−1^.

Besides that, functionalization of nanocellulose acetate with metal oxide (hydroxyapatite) had shown a very high adsorption efficiency towards heavy metals. The study by Hamad *et al.* (2020)^122^ strongly observed that the CNF functionalized with hydroxyapatite was significantly adsorbed Pb(ii) and Fe(iii) ions from simulated wastewater. It was found that, about 99.7% (46.93 mg g^−1^) and 95.46% (45.83 mg g^−1^) of Pb(ii) and Fe(iii) ions were adsorbed within 35 and 40 minutes, respectively. The adsorption process was found to obey a pseudo-second-order and Freundlich models. The desorption efficiency of the developed adsorbent was not reported in their study.

Table 8 summarizes the previous work on functionalized nanocellulose for the adsorption of heavy metals. By comparing to Table 3, it can be seen that the adsorption capacity of functionalized nanocellulose against heavy metals is comparable or has even better performance than the other types of available adsorbents. For example, the adsorption capacity of 2,2,6,6-Tetramethyl piperidin-1-yl-oxyl functionalized CNC against Cu(ii) was 339 mg g^−1^, which was higher than the adsorption capacity of thiol functionalized activated carbon (87.7 mg g^−1^).

**Table 8:** Adsorption capacity of heavy metals by nanocellulose

Type of nanocellulose	Functionalization	Heavy metal	Maximum adsorption capacity (mg g^−1^)	References
CNC	2,2,6,6-Tetramethylpiperidin-1-yl-oxyl	Ag(i)	0.86	115
CNC	2,2,6,6-Tetramethylpiperidin-1-yl-oxyl	Cu(ii)	339	115
CNC	2,2,6,6-Tetramethylpiperidin-1-yl-oxyl	Fe(ii)	416	115
CNC	2,2,6,6-Tetramethylpiperidin-1-yl-oxyl	Fe(iii)	416	115
CNC + CNF	2,2,6,6-Tetramethylpiperidin-1-yl-oxyl	Ag(i)	0.87	115
CNC + CNF	2,2,6,6-Tetramethylpiperidin-1-yl-oxyl	Cu(ii)	374	115
CNC + CNF	2,2,6,6-Tetramethylpiperidin-1-yl-oxyl	Fe(ii)	456	115
CNC + CNF	2,2,6,6-Tetramethylpiperidin-1-yl-oxyl	Fe(iii)	456	115
CNF	2,2,6,6-Tetramethylpiperidine-1-oxyl, trimethylolpropane-tris-(2-methyl-1-aziridine)propionate and polyethyleneimine	Cu(ii)	485	104
CNC	Polyethyleneimine and glutaraldehyde	As(v)	255	116
CNF	Polyethyleneimine	Cu(ii)	175	137
CNF	Polyethyleneimine	Pb(ii)	357	137
CNC	Carboxyl	Pb(ii)	64	117
CNF	Thiourea	Pb(ii)	554	118
CNC	Bentonite	Cd	2750	120
CNC	Bentonite	Co	917	120
CNC	Bentonite	Cu(ii)	1938	120
CNC	Sodium itaconate	Pb(ii)	85	139
CNF	Phosphate groups	Cu(ii)	20	140
CNF	MnFe_2_O_4_ nanoparticles	Cu(ii)	74	141
CNF	Hydroxyapatite	Co(ii)	25	142

## 7. Nanocellulose as an adsorbent of dyes

**Fig. 6:**
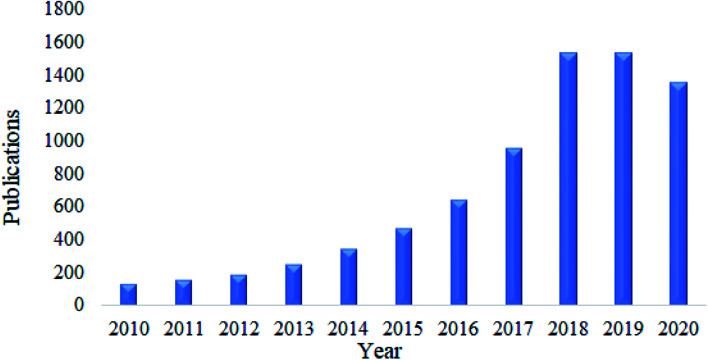
Total number of publications related to nanocellulose as an adsorbent of dyes.

The development of nanocellulose for the adsorption of dyes has also received much attention. Based on a survey done using Google Scholar and the keyword “nanocellulose as adsorbent of dyes”, the total number of publications found kept increasing over this past ten years as shown in Fig. 6. In this section, the adsorption capacity and desorption efficiency of several developed functionalized nanocellulose towards dyes as shown in Table 7 were reviewed. The adsorption capacity results reported here were compared with the currently available adsorbents for dyes as shown in Table 4.

As shown in Table 7, Yang *et al.* (2016)^123^ in their study successfully synthesized a bifunctional hairy CNC with carboxymethylated chitosan. The developed adsorbent was evaluated on its ability to remove methylene blue dyes at room temperature. It was found to achieve a maximal adsorption capacity of 785 mg g^−1^ and the dye concentration equilibrium fitted well with the Langmuir Isotherm with excellent reusability also demonstrated. The desorption study of the developed adsorbent was carried out by using hydrochloric acid and sodium hydroxide *via* shaker method. The first cycle of regenerated adsorbent showed decreasing on adsorption capacity around 86.2% and the removed amount of contaminant was constant at 188 mg g^−1^ after five subsequent cycles (83.5%).

In comparison to the above findings, Abhishek *et al.* (2017)^105^ in their study which also evaluated the adsorption efficiency of unfunctionalized nanocellulose towards methylene blue. Based on the results obtained, the unfunctionalized nanocellulose achieved an adsorption capacity of 35 mg g^−1^ which was lower than that found with the functionalized form reported by Yang *et al.* (2016).^123^ Interestingly, they found that the nanocellulose can be used repetitively for up to 6 cycles of the adsorption and desorption process.

The use of electrosterically stabilized CNC to remove the same dyes was discovered by Mandana *et al.* (2020).^124^ Adsorbent beads of sodium alginate and CNC were fabricated. The electrosterically stabilized CNC produced had a high adsorption capacity (1250 mg g^−1^) towards methylene blue. Other factors such as the effect of pH, temperature and ionic strength were also studied to evaluate its adsorbent performance. The capacity of the adsorbent for methylene blue removal decreased in presence of salts due to ion competition, while the capacity of the adsorbent increased in high pH because of deprotonation of the hydroxyl group. The desorption study was done by washed with ethanol and 1 M HCl. Dye uptake capacity decreased for each time of desorption cycle, but it appears to be levelling off at higher desorption cycles due to irreversible adsorption.

Aside from these studies, Liqiang *et al.* (2015)^125^ fabricated an adsorbent of nanocellulose and polyvinylamine (PVAm). This adsorbent was found to effectively remove Congo red 4BS, Acid red GR and reactive Light-yellow K-4G with a maximum adsorption capacity of 869.1 mg g^−1^, 1469.7 mg g^−1^ and 1250.9 mg g^−1^, respectively. At a lower pH, causing protonation of amine groups, resulted in a greater positive charge on the surface of the adsorbent. Thus, it would enhance anionic dye removal by increasing the electrostatic attraction between protonated amines and negatively charged sulphite groups on dyes. However, the desorption efficiency of the developed adsorbent was not reported in their study.

Shahnaz *et al.* (2020)^126^ prepared a functionalized CNC with polypyrrole. RSM analysis was then utilised to determine the optimum removal of Congo red dye. Based on the results obtained, efficient removal of Congo red dye was determined to be 85%. The equilibrium of the dye concentration also fitted well with the Langmuir and Freundlich Isotherm. At pH 2, the biosorption of Congo red was reported highest. The desorption efficiency of the developed adsorbent was not reported in their study.

**Fig. 7:**
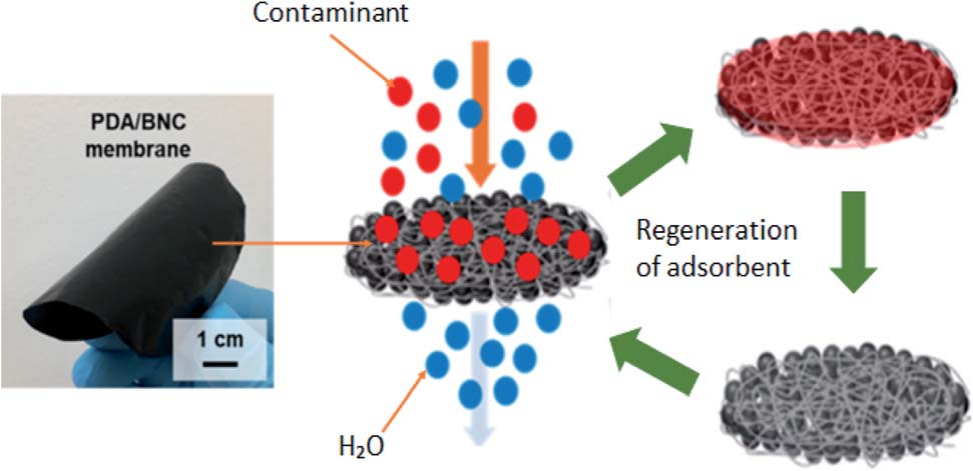
PDA/BNC adsorbent for the removal of organic dyes. Reproduced and adapted from ref. 127 with permission from American Chemical Society, copyright 2019.

BNC has also tremendous performance as an adsorbent. Hamed *et al.* (2019)^127^ investigated a novel adsorbent based on BNC incorporated with polydopamine (PDA) for the removal of dyes as is illustrated in Fig. 7 below. The PDA adsorbent was used as a catalytically active substrate for the removal of universal dyes. Performance as an adsorbent was evaluated using a simple filtration method either separately or simultaneously at room temperature within a range of pH 4–7. The results indicated that the PDA/BNC adsorbent effectively removed Rhodamine (R6G) and methylene blue (MB) dyes with excellent reusability (about 90% of initial adsorption capacity was retained after 10 cycles of filtration). However, the PDA adsorbent was not suitable for the removal of negatively charged contaminants such as Methyl Orange (MO). Besides that, the stability studies of PDA/BNC membrane were carried out by extensive sonication for 5 h and agitation for 30 days. No noticeable signs of disintegration of PDA particles inside the BNC network. The desorption of PDA/BNC membrane was capable to retain ∼81% of initial efficiency in removing the pollutants.

Later on, Hamed *et al.* (2020)^143^ continued their study into the removal of neutral dyes using BNC loaded with mesoporous PDA (mPDA) nanostructure and palladium (Pd) nanoparticles. Similar to PDA, Pd was used as a catalytically active substrate for the removal of universal dyes. Functionalization was done through mPDA nanoparticles being embedded into BNC during its growth process to create mPDA-BNC and then, *in situ* growth of Pd nanoparticles on mPDA-BNC was done. Due to the higher porosity of this adsorbent, the water flux of the adsorbent was significantly higher which then effectively removed anionic (methyl orange), cationic (methylene blue) and neutral (4-nitrophenol) dyes in a wide range of pH (4–9). The desorption study of the developed adsorbent was carried out by washing it with water and alcohol mixture and reusing it. The adsorbent retained their removal efficiency above 96% even after 5 cycles of desorption steps. Furthermore, in order to explore if the removal happens preferentially for certain dyes, a cocktail containing methyl orange, 4-nitrophenol and neutral dyes, and methylene bule was filtered through a regenerated adsorbent. All the dyes were effectively removed from the solution.

Gopakumar *et al.* (2018)^128^ introduced a new protocol for the surface treatment of CNF through a solvent-free procedure using Meldrum's acid as an esterification agent which would then be able to remove crystal violet dye. The developed adsorbent efficiently removed 99% of crystal violet dyes and Fe_2_O_3_ nanoparticles from water. The adsorption capacity of this developed adsorbent was 3.984 mg g^−1^, which was two to three times higher than that of unfunctionalized CNF due to the high electrostatic attraction between the positively charged crystal violet dyes and the negatively charged carboxylate groups in this modified CNF. The desorption efficiency of the developed adsorbent was not reported in their study.

The development of magnetic functionalized nanocellulose has also shown interesting potential for dye removal. Nasim *et al.* (2020)^129^ developed an adsorbent from CNF with magnetic nanoparticles incorporated. Due to its high magnetic behavior properties (38.5 emu g^−1^), 94.9% of Rhodamine B (RhB), an organic dye, was successfully removed in 300 min at room temperature. The removal ability of dye after few cycles was decreased due to decreasing amount of Fe_3_O_4_ in the membrane.

Besides that, several research have discovered the potential of CNC functionalized with metal oxides for removal of dyes.^130,144,145^ Hamad *et al.* (2018)^130^ had developed a carbon–phosphorus–titanium composites and used for the adsorption and photodegradation of Orange G dye. CNC was used as a carbon precursor. The efficiency of developed adsorbent in the removal of the Orange G dye in solution by adsorption and photocatalysis was evaluated. Maximum removal of Orange G dye was achieved after 20 min and composites with lower titanium-content presented a better adsorption. This is because, composites with low titanium over carbon ratio present a larger carbon phase, which has a higher affinity for Orange G dye in solution. Moreover, carbon–phosphorus–titanium composites with anatase titanium dioxide nanoparticles and large surface areas seem to be the most active photocatalysts for Orange G degradation under UV irradiation. The desorption efficiency of the developed adsorbent was not reported in their study.

Table 9 summarizes several previous works on functionalized nanocellulose which focused on dye adsorption. Each functionalization depends on a range of pH that results in various orders of maximum adsorption capacity and each taking different equilibrium contact times. By comparing with the currently available adsorbents for dye removal as listed in Table 4, the nanocellulose adsorbents that are reported on in this section have a comparable or even better performance with regards adsorption capacity towards dyes. For example, the adsorption capacity of carboxylmethyl functionalized CNC against methylene blue is 785 mg g^−1^, which is higher than the adsorption capacity of activated carbon which is only between 90 ∼ 252 mg g^−1^.

**Table 9:** Adsorption capacity of dyes by nanocellulose

Type of nanocellulose	Functionalization	Dye	Maximum adsorption capacity (mg g^−1^)	Reference
CNC	Carboxymethyl	Methylene blue	785	123
CNC	—	Methylene blue	35	105
CNC	Polyvinylamine	Congo red 4BS	869	125
CNC	Polyvinylamine	Acid red GR	1470	125
CNC	Polyvinylamine	Reactive light-yellow K-4G	1251	125
CNF with chitosan	Poly(hydroxyalkanoate)	Congo red	435	146
CNC	Fe_3_O_4_ ionic liquid	Congo red	131	147
CNC	—	Hydroxynaphtol blue	0.17 (mmol g^−1^)	148
CNC	—	Congo red	0.16 (mmol g^−1^)	148
CNF	Oxalic acid	Methylene blue	192–430	149
CNC	Magnetic	Methylene blue	60	150

## 8. Nanocellulose as an adsorbent of organic oils

**Fig. 8:**
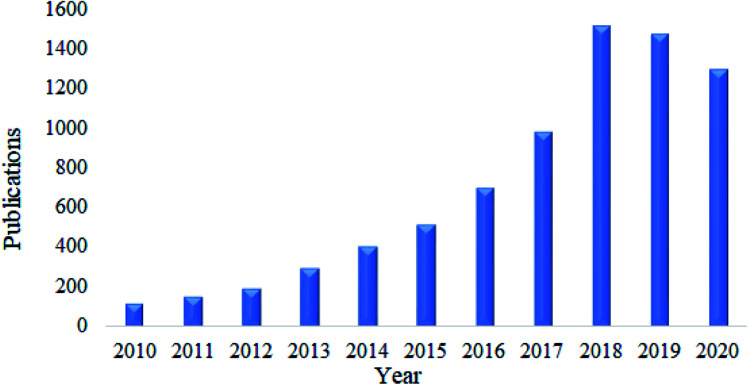
Total number of publications related to nanocellulose as an adsorbent of organic oil contamination.

Nanocellulose is also a fascinating adsorbent for organic oils. Similar to heavy metals and dyes, research related to this area has received much attention. Based on a survey done using Google Scholar using the keywords “nanocellulose as adsorbent of organic oils”, the total number of publications have kept increasing over the past ten years, and this is illustrated in Fig. 8. In this section, the adsorption capacity and desorption efficiency of several developed functionalized nanocellulose towards organic oils as shown in Table 7 were reviewed. The adsorption capacity results reported here were compared with the currently available adsorbents for organic oils as shown in Table 5.

**Fig. 9:**
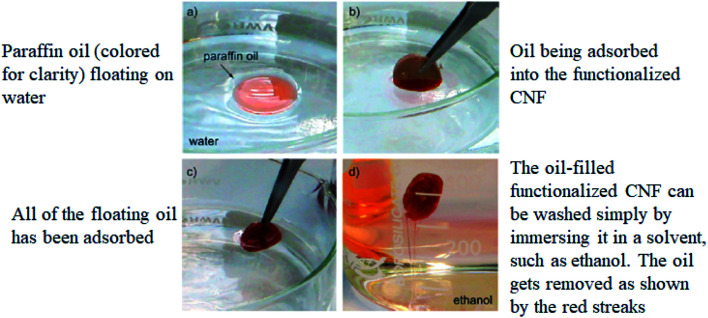
Oil spill removal from water. Reproduced and adapted from 131 with permission from American Chemical Society, copyright 2011.

In 2011, Korhonen *et al.*^131^ had functionalized the CNF with titanium dioxide for the adsorption of paraffin oil from water. This functionalized CNF demonstrated highly selective adsorption of nonpolar liquids and oils when compared to water as shown in Fig. 9 below. The amount of adsorbed paraffin oil was approximately 700 mg cm^−3^, with a selectivity of about 30 : 1 wt/wt compared to water, whereas for the same sample, water adsorption was approximately 24 mg cm^−3^. The desorption study showed that the adsorption capacity was not deteriorated and the dry weight of the developed adsorbent did not change when it was reused multiple times. Moreover, the developed adsorbent can be simply incinerated along with the absorbed oil.

**Fig. 10:**
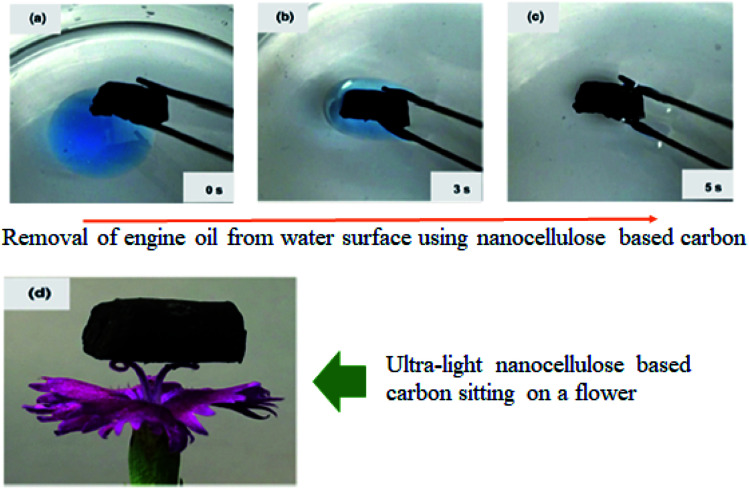
Sponge–like nanocellulose based-carbon adsorbent. Reproduced and adapted from ref. 132 with permission from Springer Nature, copyright 2014.

Another interesting work was that by Meng *et al.* (2015)^132^ where a sponge–like nanocellulose based-carbon adsorbent as shown in Fig. 10 was developed for oil adsorption. This adsorbent exhibit hydrophobic behavior, 99% porosity with ultra-light density (0.01 g cm^−3^), fast adsorption rate and reusability. Additionally, this functionalized nanocellulose had higher adsorption capacity towards a variety of oils such as canola oil, diesel oil, paraffin and pump oil. This was due to the 3D-network structure of the functionalized nanocellulose which is a key point for assuring electromechanical properties and large surface energy. Analysis showed that the highest normalized adsorption capacity (86 g g^−1^) was for paraffin oil. To investigate the desorption capability of the developed adsorbent, the oil sorption experiments were repeated after the oil-soaked samples were thoroughly cleaned in ethanol and dried. The developed adsorbent was found to retain the same oil absorption capacity after ten desorption cycles.

Chin *et al.* (2014)^151^ developed a hydrophobic, magnetic, selective and highly porous magnetic Fe_3_O_4_ functionalized nanocellulose adsorbent by the simple and fast sol–gel method. The developed adsorbent nanocellulose was able to adsorb paraffin oil by up to 28 times (28 g g^−1^) of its weight within 10 min and could be easily removed and recovered from the water surface by an external magnet. Moreover, this developed adsorbent has several advantages of ease of preparation, low-cost materials, magnetically retrievability, as well as high oil absorption capacity desorption efficiency. The desorption study had showed that this developed adsorbent could be either reused after washing with ethanol, or incinerated with the absorbed oil.

**Fig. 11:**
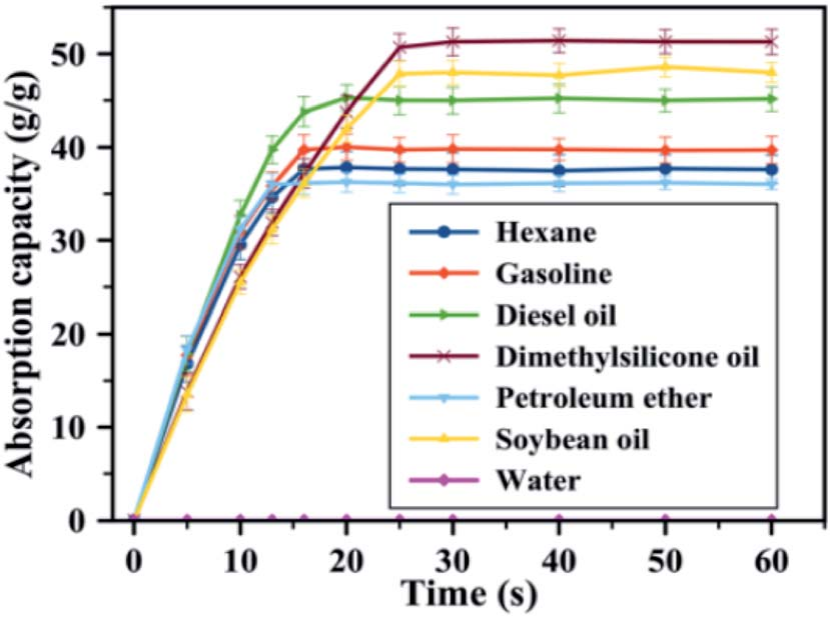
The absorption capacities of the developed adsorbent for several oily substances. Reproduced and adapted from ref. 133 with permission from Elsevier, copyright 2017.

The magnetic/silanized ethyl nanocellulose was developed by Lu *et al.* (2017).^133^ The magnetic silanized ethyl nanocellulose showed good adsorption capacity of oils (*n*-hexane, gasoline, diesel oil, dimethylsilicone oil, petroleum ether and soybean oil). Furthermore, the developed adsorbent exhibited high separation efficiency and good mass absorption capacity (37 to 51 times of their own weight) for a wide variety of oils. Fig. 11 shows the adsorption capacity of developed adsorbent towards several oils. The adsorption capacity depended not only on the density but also viscosity and surface tension. For example, although soybean oil has a higher density than dimethylsilicone oil (0.94 *vs.* 0.90 g cm^−3^), its absorption poorer. This could be explained by the higher viscosity (20 *vs.* 0.82 mPa s) and surface tension (31.2 *vs.* 21.3 mN m^−1^) of soybean oil that of dimethylsilicone oil. The desorption study was showed the absorption capacity of the developed adsorbent decreased slightly to 87.6% of its initial value, indicating a highly stable absorption and good recyclability.

In addition, nanocellulose possesses a perfect 3D skeleton and has interconnected pores similar to a honeycomb. This form of nanocellulose was synthesized by Zhang *et al.* (2016).^134^ The developed adsorbent demonstrated excellent selectivity for oils. The adsorption capacity is up to 300% and 99% of its weight and volume respectively. Furthermore, this adsorbent has stable super hydrophobic and super oleophilic properties in a strong mechanical abrasion resistant configuration. Interestingly, the adsorbent can be reused for up to 30 cycles.

A superhydrophobic and superoleophilicity CNF adsorbent was also discovered by Phanthong *et al.* (2018).^135^ In their study, stearoyl chloride was functionalized with CNF. The adsorption capacity of the developed adsorbent was tested against a variety of oils and other compounds which included dichloromethane, silicon oil, toluene, vacuum pump oil, ethanol, acetone, *n*-octane, and *n*-hexane. The adsorption capacity towards silicon oil and vacuum pump oil was 35 g g^−1^ and 39 g g^−1^, respectively. Moreover, the superhydrophobic nanocellulose sponge could be easily recovered by simple squeezing and could be reused for at least 10 cycles with sustained high separation efficiency.

Recently, Gu *et al.* (2020)^136^ functionalized the CNF with oleic acid and nanomagnetite (Fe_3_O_4_). The oleic acid and Fe_3_O_4_ act as hydrophobic and magnetic agent, respectively. The adsorption capacities of this developed adsorbent towards ethyl acetate, cyclohexane, and vacuum pump oil were 56.32, 68.06, and 33.24 g g^−1^, accordingly. In addition, this developed adsorbent demonstrates an excellent magnetic responsivity and can be easily recycled by a permanent magnet after adsorption.

Table 10 summarizes several previous works on functionalization on nanocellulose focused on organic oils adsorption. Interestingly, through comparison with the currently available adsorbents for organic oil removal as shown in Table 5, it can be seen that nanocellulose possesses an excellent and higher adsorption capacity as compared to the other types of adsorbents. For example, the adsorption capacity of carbon functionalized CNF towards diesel is 785 mg g^−1^, which is higher than the adsorption capacity of polyurethane which is only 46.98 mg g^−1^.

**Table 10:** Adsorption capacity of oils by nanocellulose

Type of nanocellulose	Functionalization	Oil	Maximum adsorption capacity (g g^−1^)	References
CNF	Stearoyl chloride	Silicon	35	135
CNF	Stearoyl chloride	Vacuum pump	39	135
CNF	Carbon	Paraffin	86	132
CNF	Carbon	Diesel	74	132
CNF	Carbon	Canola	74	132
CNF	Carbon	Pump	54	132
Cellulose	Methyltrimethoxysilane	Motor	95	152
CNF	Titanium tetraisopropoxide/Fe_3_O_4_	Paraffin	28	151
Cellulose	Fe_3_O_4_/silanized	Petroleum ether	38	133
Cellulose		Peanut	35	153
CNF	Oleic acid and nanomagnetite	Cyclohexane	68	136
		Ethyl acetate	56	136
		Vacuum pump	33	136

## 9. Challenges and future directions

The usefulness of nanocellulose and its characteristics as an adsorbent in chemical contaminants remediation is a promising and exciting area of current and future research. Several recent developments in the application of nanocellulose as adsorbent were reviewed. Functionalization of nanocellulose using a variety of functional groups is a key factor towards successful enhancement of its adsorption capacity against numerous chemical contaminants which include heavy metals, dyes, and organic oils. Most of the functionalized nanocellulose adsorbents developed as reviewed in this manuscript showed better performance than other commercially available adsorbents.

Although the effectiveness of nanocellulose as a new biobased adsorbent for a broad range of contaminants has been demonstrated through different works, several improvements are still needed. Most of the adsorption studies done are limited to batch-scale only, and few works have investigated the adsorption properties under dynamic conditions and more specifically for the treatment of industrial wastewater. The batch mode adsorption could be transferred to adsorption columns filled with nanocellulose in the form of a porous structure so that this continuous filtration can be considered in real water treatment. Additionally, more research is needed to generate hybrid structures at nanoscales on the surface of nanocellulose that would be likely to interact with different species so that it becomes possible to develop composite adsorbents capable of adsorbing multiple chemical species at a time.

Moreover, some challenges regarding nanocellulose have also been identified. Nanocellulose requires a high production cost, especially at industrial levels. The energy consumption related to the production of nanocellulose is still an issue that hampers the scale-up production of nanocellulose. However, to the best of our knowledge, progress has been accomplished in this area, and numerous pilot-scale production facilities have now become available worldwide. Another issue is the biodegradability of nanocellulose which needs to be considered. Not all research as presented here had studied on the biodegradation of the developed adsorbents. Therefore, future studies must focus on the lifespan of nanocellulose as an adsorbent. Many factors influence the degradability of nanocellulose such as types of water and the presence of certain microbes that are able to degrade the cellulose after use. These studies should look at nanocellulose in a life cycle manner, from production, use and finally with regards its disposal. With its source being from natural and mostly waste materials from agricultural activities, the evolution of its use to manage contamination in the environment leads its promise as a bioproduct. To close the loop, further investigation into its degradation in a green manner after use is still needed.

## Conflicts of interest

There are no conflicts of interest to declare.

## Supplementary Material
